# The Fungal Microbiome in the Vineyard Ecosystem Plays a Key Role in Shaping the Regional Characteristics of Wine

**DOI:** 10.3390/foods14071211

**Published:** 2025-03-30

**Authors:** Chunyan Bai, Yuan Yao, Hua Wang, Hua Li, Ruteng Wei

**Affiliations:** 1 College of Food Science and Engineering, Shanxi Agricultural University, No. 1, Mingxian South Road, Jinzhong 030801, China; 20210707628@stu.sxau.edu.cn (C.B.); 20220707807@stu.sxau.edu.cn (Y.Y.); 2 Beijing Hongxing Liuquxiang Co., Ltd., Liuquxiang Branch Company, Industrial Zone, Qixian, Jinzhong 030900, China; 3 College of Enology, Northwest A&F University, No. 22, Xinong Road, Yangling 712100, China; wanghua@nwafu.edu.cn (H.W.); lihuawine@nwafu.edu.cn (H.L.)

**Keywords:** wine, microbial diversity, regionality, metabolite profiles, random forest, partial least squares path modeling

## Abstract

The regional characteristics of wine are shaped by the synergistic effects of vineyard climate conditions, soil microbial microorganisms, soil properties, and grape must microorganisms; however, their role in shaping regional wine quality is still poorly understood. In this study, soil, grape must, and fermentation samples were collected from Cabernet Sauvignon vineyards in five regions of China. High-throughput sequencing technology was used to analyze the microbiota, and Headspace-Solid Phase Microextraction-Gas Chromatography-Mass Spectrometry (HS-SPME-GC-MS) was used to determine the wine metabolite profile. The results showed that the wine metabolite profiles from different vineyards were significantly different and could be distinguished by their volatile compounds, with each vineyard possessing unique characteristic metabolites. The geographical origin of vineyards significantly influenced the microbial diversity of both soil and winery environments. Although the microbiota changed during fermentation, regional microbial signatures were preserved at the end of fermentation. The random forest model indicated that fungal diversity and weather are key predictors influencing wine regionality, with fungal diversity in grape must having the greatest impact. Partial least squares path modeling further revealed that fungal diversity in grape must had the most significant impact on wine metabolite profiles, followed by weather and then soil fungal diversity. In contrast, soil properties and soil bacterial diversity had weaker effects on these profiles and were significantly influenced by the weather. Overall, this study provides a novel perspective for understanding the mechanisms underlying wine regionality and clarifies the key role of microorganisms, particularly fungal communities, in shaping wine regionality.

## 1. Introduction

Wine is a global economic product with significant socio-cultural value. The unique sensory characteristics of wines from different regions are referred to as “*terroir*”, which is defined in the wine industry as a collection of important product features [1]. Regional variations in “*terroir*” are predominantly manifested through the physiological adaptations of grapevines to their local environments. It is well established that soil properties (such as soil type, texture, and nutrient content), climatic factors (including temperature, precipitation, and sunlight exposure), landscape features, and vineyard management and winemaking techniques collectively shape the sensory profiles of wines from distinct regions, thus defining the concept of *terroir* [2]. Wines produced from the same grape variety exhibit regional diversity, which drives their market appeal, price differentiation, and consumer demand [3]. Nevertheless, the specific factors from vineyards and wineries that shape regional wine quality characteristics remain unclear.

As a fermented natural product, wine relies heavily on vineyard conditions as a pivotal factor in shaping microbial community quality, particularly in spontaneous fermentations, where exogenous yeasts are not introduced. The involvement and action of vineyard microbiota directly influence the types, quantities, and transformation processes of aroma compounds in wine, ultimately shaping its inherent quality and style [4]. Grapevines harbor a diverse array of microorganisms, including bacteria, filamentous fungi, and yeasts, which are present on the grape surface, within the vine, and in the soil [2]. They play a crucial role in the vineyard ecosystem, interacting with plants to influence their growth and health, grape development, and wine quality [5]. For example, yeast communities associated with grapes are an important component of the grape-wine system and contribute to the formation of *terroir*. Increasing evidence suggests that the microbiota associated with grapes and wine exhibit nonrandom geographical patterns [6,7,8]. Many studies have investigated the link between microbial communities in grape must and the chemical profiles of wine. For example, the metabolite profiles of wines and the microbiota in grape must can distinguish viticultural regions and individual vineyards, while geographically distinct *Saccharomyces cerevisiae* populations in grape must drive the metabolite uniqueness of the resulting wines [9,10,11]. However, the impact of soil microbiota on wine metabolite profiles has not yet been investigated.

Soil, as a key element of *terroir*, plays a fundamental role in shaping the characteristics and flavors of wine through its composition and structure in vineyard settings. Vineyard soils provide essential water and nutrients to grapevines, and soil type and characteristics significantly impact vine growth and development [3]. Microbial activity at the rhizosphere-soil interface can influence wine quality by modifying grape fruit chemistry. For instance, nitrogen uptake can affect the distribution of resources between plant biomass and fruit development, as well as the synthesis of secondary metabolites in grapes [12]. The nitrogen content in grapes provides nutrition to the microbiota during fermentation and is thus associated with yeast vitality and metabolite production during the fermentation process [13]. Soil microorganisms can decompose organic matter and activate plant defense responses, thereby affecting the flavor and quality of grapes and the resulting wines [14]. In addition to other factors, climate is the most critical environmental factor in viticulture, influencing the growth and persistence of indigenous microorganisms in both time and space, as well as determining wine styles, regional characteristics, and wine quality [1]. Microorganisms from vineyards enter the winery along with grapes and grape must; thus, the influence of environmental conditions is ultimately reflected in the microbiota during wine fermentation. However, the comprehensive impact of wine regionality, soil properties, and microbial diversity on wine metabolites has not yet been fully investigated.

To address this issue, we first employed high-throughput sequencing technology to characterize the microbiota in soil, grape must, and fermentation samples from five geographically distinct Cabernet Sauvignon vineyards in China. Subsequently, metabolite profiling of the resulting wines was performed using Headspace-Solid Phase Microextraction-Gas Chromatography-Mass Spectrometry (HS-SPME-GC-MS). Finally, random forest and partial least squares path modeling were applied to elucidate the relationships between soil and wine microbiota, abiotic factors (weather and soil properties), and wine regionality, thereby identifying the key factors influencing wine regionality.

## 2. Materials and Methods

### 2.1. Sampling

In this study, representative Cabernet Sauvignon vineyards in the Yinchuan, Wuzhong, Wuwei, Changli, and Jinzhong wine—producing regions of China in 2022 (named YC, WZ, WW, CL, and JZ, respectively) were selected as the research objects. The selected vineyards cover arid, semi—arid, and temperate monsoon climate regions, which can reflect the growth characteristics of grapes under different ecological conditions (see Figure S1 in the Supplementary Materials). All the vineyards were commercially managed using similar chemical viticulture methods. For example, fungicides such as sulfur powder, Bordeaux mixture, Mancozeb and Iprodione were used to control powdery mildew and downy mildew. Vineyard conditions (including elevation, annual average temperature, annual precipitation, Sunshine duration, aspect, soil characteristics, and cover crops) are presented in Supplementary Table S1. The distances between the vineyards ranged from 20 to 1500 km. Each vineyard was equipped with an independent weather station, from which weekly measurements of sunshine hours (SSH), precipitation (PRE), mean low temperature (MLT), mean high temperature (MHT), mean temperature (MT), mean ground temperature, and relative humidity (RH) were extracted for the growing season (April to October).

In each vineyard, during the harvest period from September to October, three sampling sites representing the cultivated areas of the vineyard were selected. Soil samples were collected at a depth of 0 to 10 cm, 30 cm away from the grapevines (three subsamples from each sampling site were combined to form a composite sample). All samples (n = 15) were stored in sterile bags, transported on ice, and stored at −80 °C until processing.

Meanwhile, healthy grapes were manually harvested for fermentation. Prior to being filled with grapes, we cleaned and disinfected the fermentation tank and space environment. The grapes were crushed and loaded into the tank, filling 75–80% of the 1 m^3^ tank capacity. During grape crushing, tank filling, and throughout fermentation, no SO_2_ or yeast nutrients were added. Fermentations were conducted at each winery under a standardized protocol by omitting commercial yeast and bacterial inoculations and relying exclusively on spontaneous fermentation processes. During fermentation, the fermentation temperature was controlled at 24–26 °C, and the must was pumped over three times per day. The initial concentrations of sugars and acids in the grape must were similar across all samples (Table S1). Sampling was conducted at five stages of fermentation to characterize the microbiota: day 1 (A, destemmed, crushed grapes prior to fermentation), day 3 (B, when the density decreases by 0.003–0.005), day 5 (C, when the density decreases by 0.03–0.04), day 9 (D, when the density decreases by 0.07–0.08), and day 15 (E, when the density is less than 1.0 and remains stable). Samples were collected in triplicate from the fermentation tanks (from the top, middle, and bottom). All samples (n = 75) were immediately frozen at the winery upon collection, transported to the laboratory on dry ice, and stored at −20 °C until processing.

### 2.2. Soil Analysis

Soil factor analysis was conducted to elucidate the impact of soil properties on the wine-associated microbiota and metabolite profiles. The samples were air-dried and ground through a 20-mesh sieve for pH determination. The samples were extracted with 2.5 times their weight of distilled water, and the pH was measured using a pH meter (CyberScan pH 510, Thermo Fisher Scientific, Waltham, MA, USA). The air-dried samples were ground through a 60-mesh sieve to determine total carbon (TC), total nitrogen (TN), total phosphorus (TP), total potassium (TK), and organic matter (OM) contents. A 200 mg soil sample was weighed, placed in a tin capsule, and crimped using a sample press. Total carbon (TC), nitrogen (TN), phosphorus (TP), and potassium (TK) were quantified via high-temperature combustion (1150 °C) using a Vario MACRO cube elemental analyzer (Elementar, Germany) with thermal conductivity detection (TCD). Calibration was performed using certified reference materials, and the analytical precision was maintained within a 1.5% relative standard deviation (RSD) [15]. Soil organic matter (OM) content was calculated as total carbon (TC) multiplied by 1.724, following the Walkley-Black correction, which accounts for incomplete oxidation during wet digestion [16]. The soil C/N ratio was calculated based on the contents of total carbon and total nitrogen.

### 2.3. Analysis of Volatile Compounds in Wine

VVolatile compounds were extracted using HS-SPME with slight modifications, as described by Hu et al. (2019), and then analyzed by GC–MS using the Agilent 7890A–5975C system (Agilent, Santa Clara, CA, USA) [17]. In brief, a 5 mL wine sample was added to a 20 mL glass vial containing 1 g of NaCl and 10 µL of an internal standard (16 mg/L, 2-octanol). The mixture was equilibrated at 40 °C for 15 min. A 50/30 µm DVB/CAR/PDMS fiber was then immersed in the headspace and stirred at 40 °C and 600 rpm for 30 min. The fiber was subsequently desorbed in the GC injector for 5 min at 230 °C using a DB-WAX column. Helium (99.999%) was used as the carrier gas at a flow rate of 1.5 mL/min. The gas chromatography (GC) program was as follows: 40 °C for 3 min, ramped at 4 °C/min to 160 °C, then at 7 °C/min to 220 °C, and held for 8 min. The temperatures of the GC column and the transfer line were maintained at 220 °C, and the ion source temperature was set at 200 °C. Electron ionization (EI) mass spectrometry (MS) data were acquired by scanning the mass-to-charge ratio (*m*/*z*) range of 35 to 350 at 0.2 s intervals. Volatile compounds were qualitatively identified by comparing their retention times and mass spectra with those of pure reference compounds using the NIST 17 mass spectral library. The concentrations of the compounds were calculated based on the calibration curves.

### 2.4. DNA Extraction and Sequencing

Genomic DNA was extracted from the soil and fermentation samples using the EZNA^®^DNA Kit (Omega Bio-tek, Norcross, GA, USA) according to the manufacturer’s instructions. The quality of the extracted DNA was assessed using a Qubit fluorometer and a Nanodrop spectrophotometer (Thermo Fisher Scientific, Waltham, MA, USA). Genomic DNA was submitted to Gene Denovo company for amplification and sequencing. Referring to the method of Wei et al. (2022), the ITS2 region of the ITS rRNA gene was amplified with the specific primers ITS3_KYO2F and ITS4-2409R, and the V3-V4 region of the 16S rRNA gene was amplified with the specific primers 341F and 806R [11]. According to standard operations, purified amplicons were sequenced using paired-end sequencing (Hiseq 2500, PE250) on the Illumina platform.

We use FASTP (version 0.18.0) and FLSAH (version 1.2.11) to perform quality filtering and sequence merging on the raw fastqfiles of 50 microbial samples to obtain tags. Then, according to the QIIME (version 1.9.1) Tags Quality Control process, low-quality tags were filtered to obtain high-quality clean tags. Based on the reference database, the UCHIME Algorithm is used to detect and delete the chimeras of tags to obtain effective Clean Tags. Finally, Clean Tags were assigned to the same OTUs (Operational Taxonomic Units) according to the similarity of ≥97% by using UPARSE (version 9.2.64). The tag sequence with the highest abundance was selected as the representative sequence for each OTU. For each representative sequence, the classification information was annotated using the Silva database based on the Mothur algorithm.

### 2.5. Data Analysis

Principal Coordinate Analysis (PCoA) was performed based on Bray-Curtis dissimilarity to assess the distribution patterns of microbial and metabolite samples using the ‘labdsv’ package. One-way analysis of variance (ANOVA) and multivariate analysis of variance (PERMANOVA) were used to determine whether the sample classification exhibited statistically significant differences in diversity. Linear Discriminant Analysis Effect Size (LEfSe) was used to identify the taxa that distinguished the different sample groups. The OTU table was filtered to retain only those OTUs with a relative abundance greater than 0.01%, thereby simplifying the LEfSe analysis. Significant abundance differences among categories were identified using the Kruskal-Wallis rank-sum test (α = 0.05), and the effect size for each OTU was assessed using the Linear Discriminant Analysis (LDA) score (threshold = 2.0). A supervised random forest classification model was used to identify the key determinants of wine regionality based on soil fungal and bacterial diversity (Shannon index), grape must fungal and bacterial diversity (Shannon index), soil properties, and weather conditions. The significance of each predictor was evaluated by examining the reduction in prediction accuracy, quantified by the increase in the mean squared error (MSE) between the observed values and out-of-bag predictions when the data were randomly shuffled. Partial least squares path modeling (PLS-PM) was used to analyze the direct and indirect relationships among soil fungal and bacterial diversity, grape must fungal and bacterial diversity, soil properties, weather conditions, and wine regionality. An a priori model was constructed based on established relationships among factors influencing the regional distribution of wine metabolites, with data preprocessed before model application. The path coefficients describe the strength and sign of the relationship between the two variables. The significance of the estimated path coefficients was evaluated using a 95% confidence interval. PLS-PM analysis was conducted using the ‘plspm’ package in R version 4.0.2.

## 3. Results

### 3.1. Wine Metabolite Profile Can Distinguish Vineyards

**Figure 1:**
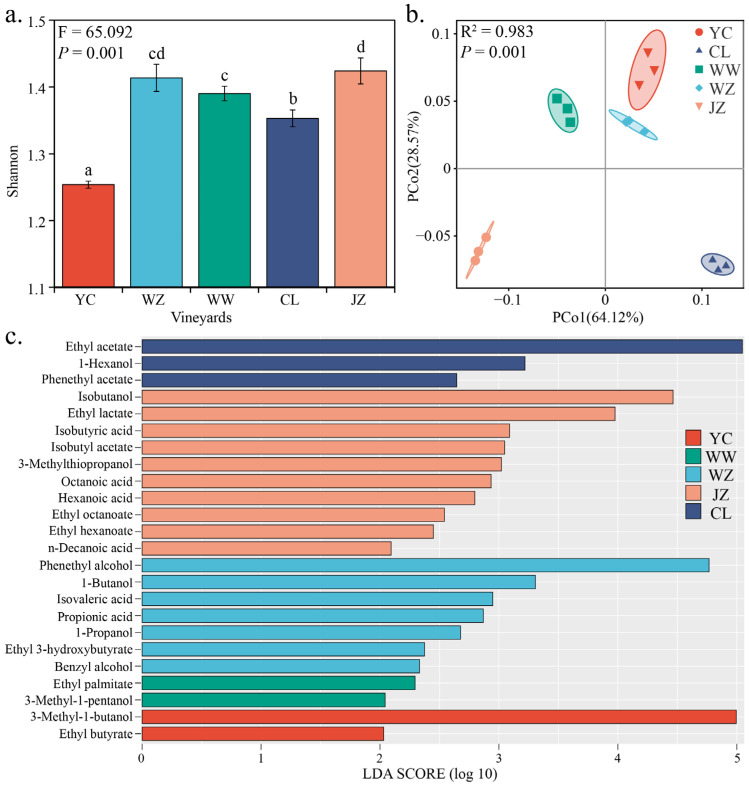
The metabolite profiles of wines from different vineyards exhibit distinct regional differences. Note: The figure shows α-diversity analysis based on the Shannon index (**a**), PCoA analysis based on Bray-Curtis distance (**b**), and characteristic metabolites (**c**) identified by LEfSe in wine. Different letters indicated the difference, with statistical significance (*p* < 0.05).

The volatile compounds of the resulting Cabernet Sauvignon wines (E stage) were analyzed using HS-SPME-GC-MS to characterize the metabolite profiles of the wines from different vineyards. A total of 70 volatile compounds were identified in these wines, of which 51 exhibited significant differences between at least two vineyards (see Table S2 in the Supplementary Material). Subsequently, α-diversity and β-diversity were used to elucidate the complexity and regionality (the differences between wines from different regions) of the wines. As indicated by the Shannon index, the α-diversity of the wine metabolite profiles varied with regional origin (Figure 1a, ANOVA, F = 65.092, *p* = 0.001). The Shannon index of the wine metabolite profiles from the JZ vineyard was the highest (1.42 ± 0.02), followed by the WZ vineyard (1.41 ± 0.02), WW vineyard (1.39 ± 0.01), CL vineyard (1.35 ± 0.01), and YC vineyard (1.25 ± 0.01); among these, the Shannon index of the WZ vineyard did not significantly differ from those of the JZ and WW vineyards (Figure 1a). Principal Coordinate Analysis (PCoA) based on Bray-Curtis dissimilarity revealed that all wine samples from different vineyards were clearly separated; the first two principal coordinates (PC) accounted for 92.69% of the total variation; PMANOVA also confirmed significant differences in the metabolite profiles of wines from different vineyards (R^2^ = 0.983, *p* = 0.001); among them, the YC samples exhibited shorter distances to the WZ and WW samples, and longer distances to the JZ and CL samples (Figure 1b); geographically, the YC vineyard is closer to the WZ and WW vineyards and farther from the JZ and CL vineyards (Figure S1). Linear Discriminant Analysis Effect Size (LEfSe) analysis further confirmed that this distribution pattern was significantly associated with the metabolite profiles of wines and vineyard locations (Figure 1c). LEfSe analysis revealed that YC had two characteristic metabolites, namely 3-methyl-1-butanol and ethyl butyrate; WW had two characteristic metabolites, namely ethyl palmitate and 3-methyl-1-pentanol; WZ had seven characteristic metabolites, including phenethyl alcohol, 1-butanol, and isovaleric acid; JZ had ten characteristic metabolites, including isobutanol, ethyl lactate, and isobutyric acid; and CL had three characteristic metabolites, namely ethyl acetate, 1-hexanol, and phenethyl acetate.

### 3.2. Geographical Origin of Vineyards Leads to Differences in Soil Microbial Diversity

To investigate the role of vineyard soil and winery microbial diversity in shaping the regional characteristics of wines, 90 samples, including soil, must, and fermenting must, were collected for the analysis of wine-associated microbiota. A total of 10,522,051 high-quality 16S rRNA sequences and 10,440,758 high-quality ITS sequences were obtained, which were clustered into 27,475 bacterial and 15,132 fungal operational taxonomic units (OTUs) using a 97% similarity threshold. Additionally, the rarefaction curves of all samples tended to be flat (Figure S2), indicating a reasonable number of samples and good OTU coverage provided by high-throughput deep sequencing.

**Figure 2:**
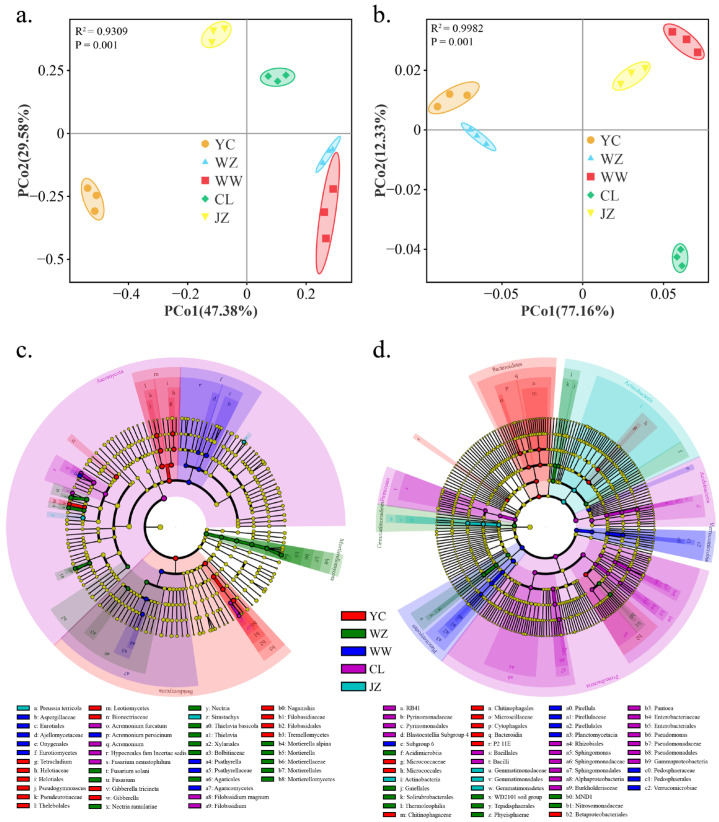
Soil microbiota from different vineyards exhibit regional differences. Note: PCoA analysis based on Bray-Curtis and weighted UniFrac distances revealed the distribution patterns of soil fungal (**a**) and bacterial (**b**) samples from different vineyards. LEfSe identified the differentially abundant fungal (**c**) and bacterial (**d**) taxa among the vineyards (LDA > 4).

At the phylum level, the dominant fungal taxa in all soil samples were *Ascomycota*, *Basidiomycota*, *Mortierellomycota* and *Mucoromycota*. Among these, the relative abundances of *Ascomycota* and *Basidiomycota* exhibited significant differences among vineyard soils (*p* < 0.05), while *Mortierellomycota* and *Mucoromycota* did not show significant differences (Figure S3a, *p* > 0.05). The dominant bacterial phylum in the collected soil samples were *Proteobacteria*, *Firmicutes*, *Actinobacteria*, *Bacteroidetes*, *Cyanobacteria*, *Chloroflexi*, *Patescibacteria*, *Planctomycetes*, *Chlamydiae*, and *Acidobacteria*; among these, the relative abundances of *Proteobacteria*, *Firmicutes*, *Actinobacteria*, *Bacteroidetes*, and *Chloroflexi* exhibited significant differences among vineyard soils (*p* < 0.05), while *Cyanobacteria*, *Patescibacteria*, *Planctomycetes*, *Chlamydiae*, and *Acidobacteria* did not show significant differences (Figure S3b, *p* > 0.05). The diversity (α-diversity, Shannon index) of soil bacteria and fungi exhibited significant differences among the vineyards (ANOVA; F_fungi_ = 6.988, *p* < 0.01; F_bacteria_ = 404.752, *p* < 0.01). Compared to bacteria, the soil fungal communities exhibited lower diversity (Table S3). At the OTU level, PCoA analyses were performed for fungi and bacteria based on Bray-Curtis distance and weighted UniFrac distance, respectively, and the results showed that soil fungal and bacterial community samples from different regions were significantly separated (Figure 2a,b). The first two principal coordinates (PC) axes explained 76.92% of the total variation for fungi and 89.49% for bacteria (Figure 2a,b). PERMANOVA also confirmed significant differences in soil microbiota among the wine regions (Fungi, R^2^ = 0.9309, *p* = 0.001; Bacteria, R^2^ = 0.9982, *p* = 0.001).

LEfSe analysis further identified the differentially abundant taxa associated with distinct vineyard soils (Figure 2c,d; Kruskal-Wallis rank-sum test, α < 0.05). In the fungal communities, the YC vineyard soil was characterized by abundant *Leotiomycetes*, *Gibberella*, and *Tremellomycetes*; the WZ vineyard soil was characterized by abundant *Mortierellomycetes*, *Fusarium*, *Nectria*, *Thielavia*, *Xylariales*, *Bolbitiaceae*, and *Agaricales*; the WW vineyard soil was characterized by abundant *Eurotiomycetes*, *Psathyrellaceae*, *Agaricomycetes*, and *Acremonium persicinum*; the CL vineyard soil was characterized by abundant *Acremonium* and *Filobasidium*; the JZ vineyard soil was characterized by abundant *Preussia terricola* and *Sirastachys* (Figure 2c). In the bacterial communities, the YC vineyard soil was characterized by abundant *Bacteroidetes*; the WZ vineyard soil was characterized by abundant *Thermoleophilia*, *Phycisphaerae*, *Nitrosomonadaceae*, and *Acidimicrobiia*; the WW vineyard soil was characterized by abundant *Planctomycetacia* and *Verrucomicrobiae*; the CL vineyard soil was characterized by abundant *Proteobacteria*, *Pyrinomonadales*, and *Bacilli*; the JZ vineyard soil was characterized by abundant *Gemmatimonadetes* and *Actinobacteria* (Figure 2d).

### 3.3. The Geographical Origin of Vineyards Leads to Differences in Winery Microbial Diversity

**Figure 3:**
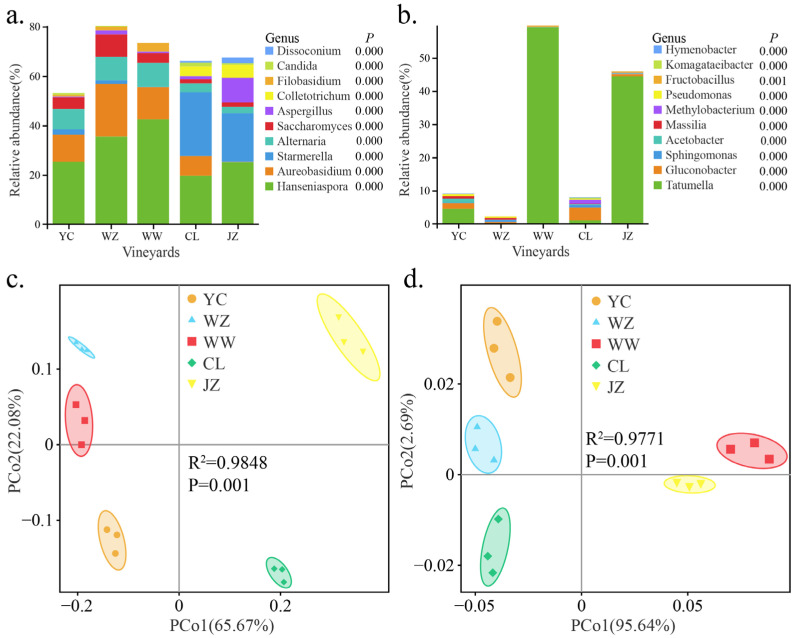
Composition and diversity of microbiota in must from different regions. Note: Composition of fungal (**a**) and bacterial (**b**) communities in must (Top 10 relative abundances; statistical significance was determined at *p* < 0.05); PCoA analysis based on Bray-Curtis and weighted UniFrac distances revealed the distribution patterns of fungal (**c**) and bacterial (**d**) samples from different regions in grape must.

In grape must, fungal communities from all regions were dominated by *Ascomycota*, primarily composed of the ubiquitous taxa *Dissoconium*, *Candida*, *Filobasidium*, *Colletotrichum*, *Aspergillus*, *Saccharomyces*, *Alternaria*, *Starmerella*, *Aureobasidium*, and *Hanseniaspora*. The relative abundances of these dominant taxa exhibited significant differences among musts from different origins (Figure 3a, *p* < 0.05). It is worth noting that the relative abundance of *Saccharomyces* varied among regions, ranging from 1.67% (CL) to 9.12% (WZ), while the relative abundance of *Hanseniaspora* ranged from 19.64% (CL) to 42.43% (WW) (Figure 3a). The bacterial communities were dominated by *Proteobacteria*, primarily composed of the ubiquitous taxa *Tatumella*, *Gluconobacter*, *Sphingomonas*, *Acetobacter*, *Massilia*, *Methylobacterium*, *Pseudomonas*, *Fructobacillus*, *Komagataeibacter*, and *Hymenobacter*, and the relative abundances of these dominant taxa exhibited significant differences among musts from different origins (Figure 3b, *p* < 0.05). The microbial diversity of fungi and bacteria in must (α-diversity, Shannon index) exhibited significant differences among regions (ANOVA; F_fungi_ = 79.155, *p* < 0.01; F_bacteria_ = 21.418, *p* < 0.01). Compared to bacterial communities, fungal communities in the must exhibited higher diversity (Table S3). At the OTU level, PCoA was performed on fungal and bacterial communities based on Bray-Curtis distance and weighted UniFrac distances, respectively. The results showed that fungal and bacterial community samples from the must were distinctly separated among the different regions (Figure 3c,d). The first two principal coordinates (PC) axes explained 87.75% of the total variation for fungi and 98.33% for bacteria (Figure 3c,d). PERMANOVA analysis also confirmed significant differences in the microbiota of musts among regions (Fungi, R^2^ = 0.9848, *p* = 0.001; Bacteria, R^2^ = 0.9771, *p* = 0.001).

**Figure 4:**
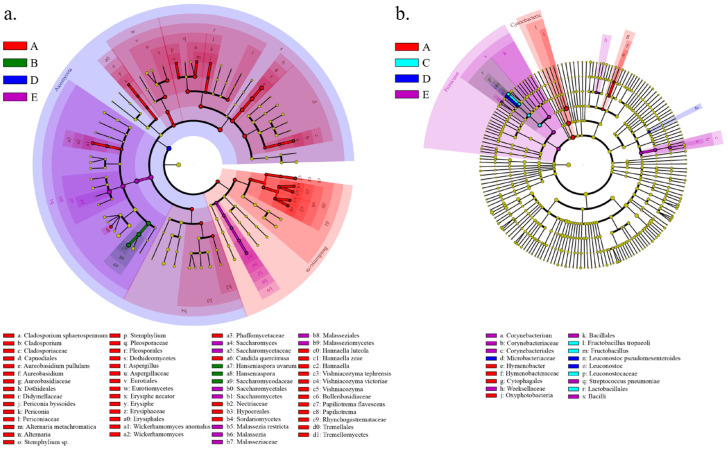
LEfSe analysis identified differentially abundant fungal (**a**) and bacterial (**b**) taxa during fermentation.

As wine fermentation progressed, fermentative populations, including *Saccharomyces*, continuously increased and became dominant, thereby reshaping the community diversity (Figure S4a,b) and composition (Figure S4c,d). Fungal species diversity significantly decreased with the progression of alcoholic fermentation (ANOVA; F = 49.413, *p* < 0.001) (Figure S4a), while bacterial species diversity showed no significant change during the early to mid-stages of fermentation but significantly increased in the later stages (ANOVA; F = 13.021, *p* = 0.023) (Figure S4b). LEfSe analysis further identified the differentially abundant taxa associated with the fermentation stages (Kruskal-Wallis test, α < 0.05) (Figure 4). For fungi, the LEfSe analysis revealed a total of 46 differentially abundant taxa at stage A, including *Dothideomycetes*, *Capnodiales*, and *Dothideales*; at stage B, 3 taxa exhibited significant differences in relative abundance, including *Hanseniaspora* and *Saccharomycodaceae*; at stage C, no differentially abundant taxa were identified; at stage D, 1 taxon, *Ascomycota*, showed significant differences in relative abundance; at stage E, 9 differentially abundant taxa were detected, including *Saccharomyces*, *Malasseziaceae*, and *Malassezia* (Figure 4a). For bacteria, the LEfSe analysis revealed a total of 6 differentially abundant taxa at stage A, including *Oxyphotobacteria* and *Hymenobacter*; at stage B, no differentially abundant taxa were identified; at stage C, 4 taxa exhibited significant differences in relative abundance, including *Fructobacillus*, *Lactobacillales*, and *Leuconostocaceae*; at stage D, 3 taxa showed significant differences in relative abundance, including *Leuconostoc* and *Microbacteriaceae*; at stage E, 8 differentially abundant taxa were detected, including *Firmicutes*, *Bacilli*, and *Weeksellaceae* (Figure 4a). In the finished wine, significant regional differences in microbial community distribution were still observed (Figure S4e,f, PERMANOVA, R^2^_fungi_ = 0.9714, *p* = 0.001; R^2^_bacteria_ = 0.8053, *p* = 0.001).

### 3.4. Multiple Factors Influence the Metabolite Profiles of Wine

**Figure 5:**
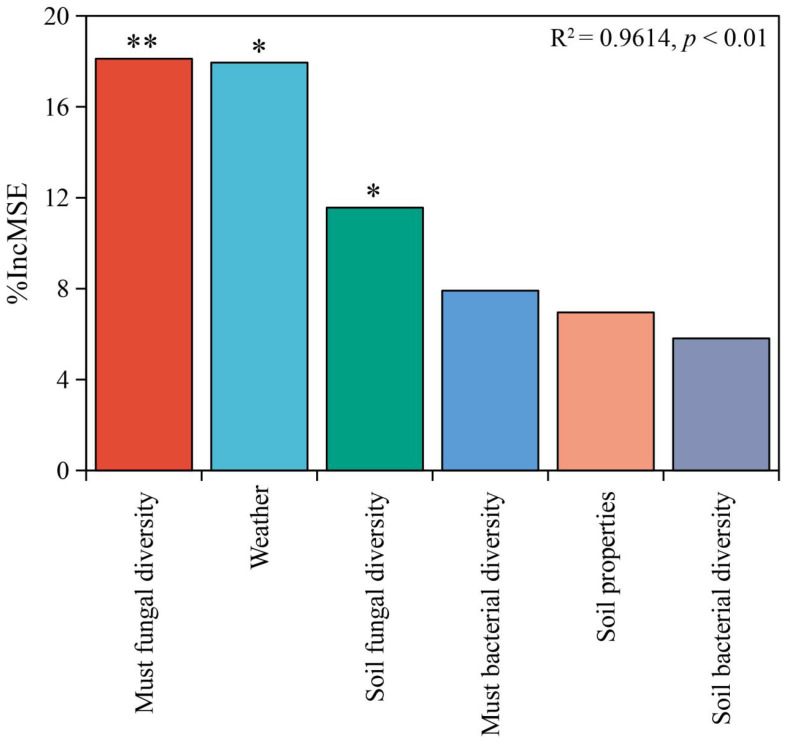
Key predictors of wine regionality. Note: The mean predictor importance from the random forest model is represented by the percentage increase in the mean squared error (MSE) associated with climate, soil properties, and microbial diversity (Shannon index) in the context of wine regionality. Significance levels: *, *p* < 0.05; **, *p* < 0.01.

In addition to the regional patterns of soil and grape must microbiota, environmental indicators of wine-producing regions also exhibited significant differences, including soil properties and weather conditions in vineyards during the growing season (April to October 2022). To elucidate the role of microbial diversity in wine regionality, a random forest model was employed to rank the importance of biotic predictors (soil and must microbial diversity) and abiotic predictors (soil and weather parameters) influencing wine regionality. Additionally, PLS-PM was used to assess whether the relationship between microbial diversity and wine regionality remains significant when accounting for multiple factors simultaneously. The random forest model (R^2^ = 0.9614, *p* < 0.01) identified must fungal diversity, soil fungal diversity, and weather conditions as significant predictors of wine regionality. Among these, fungal diversity in grape must had the greatest impact on wine regionality, followed by weather conditions and then soil fungal diversity. In contrast, bacterial diversity (in both grape must and soil) and soil properties had no significant influence on wine regionality, as evidenced by the increase in the mean square error (MSE) (Figure 5).

**Figure 6:**
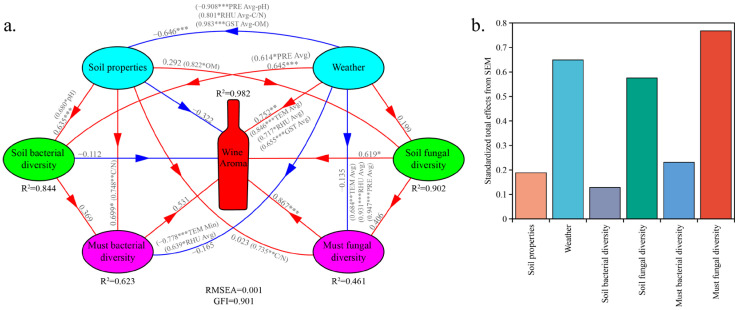
Direct and indirect effects of weather, soil properties, and microbial diversity (Shannon index) on wine regionality. Note: PLS-PM was employed to analyze the influence of the main predictive factors on wine metabolite profiles (**a**) and to derive the standardized total effects (direct and indirect effects) from the model (**b**). Climate and soil properties represent composite variables encompassing multiple observed parameters (see the Section 2). The red line with an arrow indicates a positive effect and the blue line with an arrow indicates a negative effect. Numbers adjacent to arrows are path coefficients that indicae the effect size of the relationship. The correlations between the specific parameters of the variables are indicated in parentheses. R^2^ denotes the proportion of the variance explained. Significance levels: *, *p* < 0.05; **, *p* < 0.01; ***, *p* < 0.001.

In the PLS-PM model, the Root Mean Square Error of Approximation (RMSEA) was 0.001, and the Goodness of Fit (GOF) index was 0.901, indicating an excellent overall model fit. The model explained 98.2% of the variance in the regional distribution patterns of wine metabolite profiles (Figure 6a). The model results showed that must fungal diversity had the strongest significant direct positive effect on wine metabolite profiles (R = 0.867, *p* < 0.001; Figure 6a). Secondly, weather conditions, especially TEM Avg, RHU Avg and GST Avg, had a significant direct positive effect on wine metabolite profiles (R = 0.752, *p* < 0.01); moreover, these weather variables also indirectly influenced wine metabolite profiles through their strong effects on soil and must microbial diversity (e.g., PRE Avg, RHU Avg, TEM Avg) (Figure 6a). Finally, soil fungal diversity had a significant direct positive effect on the wine metabolite profiles (R = 0.619, *p* < 0.05; Figure 6a). In addition to the aforementioned factors, must bacterial diversity also had a direct positive effect on wine metabolite profiles, although this effect was not statistically significant (R = 0.531, *p* > 0.05); soil properties and soil bacterial diversity exerted weaker negative effects on wine metabolite profiles (R_soil properties_ = −0.322, *p* > 0.05; R_soil bacterial diversity_ = −0.112, *p* > 0.05) (Figure 6a).

## 4. Discussion

### 4.1. Microorganisms as Integral Components of Wine Terroir

The present findings align with the growing body of literature highlighting the distinct biogeographical patterns of soil and must microbiota, which underpin the integration of microbiological components into *terroir* conceptualization [11,18,19]. Our results reveal significant inter-regional variations in both vineyard soil and grape must microbiota, particularly in the relative abundance of key microbial taxa. *Proteobacteria*, *Firmicutes*, *Actinobacteria*, and *Bacteroidetes* dominated the bacterial communities in soils across all sampled regions, while *Ascomycota* and *Basidiomycota* were the major fungal phyla. These observations are consistent with previous metagenomic studies that demonstrate the prevalence of these microbial groups in viticultural ecosystems [19,20,21]. Notably, inter-vineyard variability in microbial abundance was particularly pronounced for fermentative yeasts (*Saccharomyces*, *Candida*, and *Hanseniaspora*) and lactic acid bacteria (*Fructobacillus*) in grape musts, reflecting dynamic interactions between indigenous microbiota and environmental factors during grape ripening. Furthermore, previous studies have shown that distinct vineyard soil microbiomes can influence grape and wine composition, thereby shaping the wine *terroir* [4,22]. Soil serves as a critical reservoir for yeast inoculants in grape must, which may influence fermentation kinetics and metabolite production [20], thereby acting as a key determinant of the chemical profiles of wine. Our results further highlight the importance of fungal diversity in mediating these effects, particularly in yeast-dominated fermentations that drive sensory attributes.

Environmental factors (e.g., vineyard weather) and geographical characteristics of vineyards influence the microbial diversity and spatial distribution patterns of microbiota in soil ecosystems [23]. Liu et al. (2020) investigated microbial diversity in vineyard soils and grape must in South Australia, revealing significant differences in microbial community structures between these two environments attributable to seasonal or interannual variations [24]. This study also indirectly demonstrated that microbial biogeographical communities in soils and must from different vineyards are distinct. These findings are consistent with prior research on the microbial biogeography of wine and further support the notion that microorganisms are integral to *terroir* [6,18,23]. Soil bacteria and fungi can differentiate wine regions, with their distribution being influenced by soil properties and weather conditions, as confirmed by previous work [21,25]. Both fungal and bacterial diversity are strongly influenced by soil properties and weather conditions, particularly pH and PRE Avg. Soil pH affects the membrane potential and proton motive force of bacteria and fungi, thereby influencing their uptake of nutrients. Additionally, optimal pH values facilitate the cycling and transformation of essential nutrients, such as nitrogen and phosphorus, in the soil, thereby providing favorable conditions for bacterial and fungal growth [26,27]. Moderate precipitation events serve to augment soil moisture levels, which in turn create microenvironmental conditions conducive to bacterial and fungal proliferation through improved nutrient bioavailability and reduced osmotic stress [28]. It is worth noting that soil organic matter has a significant impact on soil fungal diversity. As soil organic matter can be managed through composting and conservation tillage [29], vineyard management practices have the potential to influence the wine microbiota. Additionally, the diversity of grape must fungi, which directly influences the wine metabolite profile, was indirectly affected by weather and soil properties that impact soil fungal diversity.

It is well known that the composition and structure of microbiota change during wine fermentation. In particular, fungal diversity gradually decreased during fermentation, with a notable reduction in fungal taxa, indicating that the microbial biogeographical characteristics of wines from different regions diminished during fermentation. This change was more pronounced in the fungal communities than in the bacterial communities. This is not surprising, as the growth of *S. cerevisiae* increases the fermentation rate, temperature, and ethanol concentration, and these harsh conditions have a greater impact on fungal populations than on bacterial populations [11]. The chemical environment of wine and the interactions and competition among strains within the microbiota reshape the observed microbial biogeographical characteristics [30]. Despite marked changes in microbial ecology during fermentation, microbial biogeographical patterns in grape must from different regions retained region-specific characteristics at the end of fermentation, which were further reflected in the regional metabolite profiles of wines. In the resulting wines, many volatile compounds were detected, including alcohols, esters, acids, and aldehydes, which are often secondary metabolites of microorganisms. Compounds characteristic of grape variety aromas, such as terpenes, can also be detected. During fermentation, yeasts and bacteria produce enzymes, including glycosidases, esterases, and lipases, which modify grape-derived terpenes, thereby enhancing the floral and fruity aromas of wine [31]. *Hanseniaspora* and *Candida* were identified as critical non-*Saccharomyces* yeasts during fermentation in this study, with their synergistic relationships with specific volatile metabolites being well-documented in previous research. For example, *Hanseniaspora* demonstrated strong correlations with isobutyl acetate, isoamyl acetate, ethyl hexanoate, and methyl octanoate, which are ester compounds known to enhance the s fruity aromatic characteristics of wine [17]. *Candida* can produce β-glucosidase, which may utilize glycosidic precursors of volatile compounds extracted from grapes as substrates to form geraniol, α-terpineol, and other varietal compounds, thereby enhancing the expression of the local *terroir* [11]. In this study, the model indicated that weather and soil properties have a stronger indirect influence on wine metabolite profiles through their effects on soil and must microbial diversity than through direct effects. This finding aligns with emerging theories highlighting microbial communities as critical mediators of environmental inputs in terroir expression [1], suggesting that microbe-driven biochemical transformations are the primary determinants of regional wine characteristics.

### 4.2. Mycobiota Shape the Regionality of Wine

Bacterial and fungal communities in the must showed distinct responses to regional and environmental conditions. Fungal communities exhibit significantly distinct distribution patterns at the regional scale, while bacterial differences are relatively less pronounced, consistent with the findings of Bokulich et al. (2013) [6]. Although bacteria and fungi are closely related to soil properties and influence the wine fermentation process, the impact of bacteria on wine aroma is not significant. The PLS-PM model additionally revealed that fungal diversity in must and soil had a significant impact on wine metabolite profiles. Soil fungi can migrate to grape berry surfaces via natural processes, such as rain splash and insect activity, and subsequently enter fermentation tanks during harvest [32]. During fermentation, these fungi release metabolites that influence the flavor and quality of wine. The geographical diversity of grape must yeast communities has been confirmed through the chemical attributes of wine. For example, Knight et al. (2015) demonstrated that *S. cerevisiae* populations from different regions produce distinct metabolites [10]. Additionally, Bokulich et al. (2016) reported microbial patterns associated with regional metabolite profiles and highlighted the importance of fermentative yeasts, such as *S. cerevisiae*, *Hanseniaspora*, and *Pichia* [9]. The correlation between these yeasts and metabolites can be used to predict wine composition, style, and region of origin, warranting further investigations. While fungal community diversity in soil (measured by the number of phyla detected) is lower than bacterial community diversity, it plays a more significant role in shaping the regional characteristics of wine. Soil fungal diversity, influenced by vineyard soil properties and weather conditions, in turn affects grape must fungal diversity. The yeast in the must has been shown to originate from microbial consortia on grape surfaces, with vineyard soil serving as the primary reservoir for these microorganisms [20]. One possible explanation for this is that grapevines selectively filter soil microbiota, thereby shaping the microbial consortia present in grapes and must. However, the mechanisms underlying this filtering process remain unclear. The heightened sensitivity of vineyard soil fungi may enhance grape adaptability to local environments, thereby amplifying the regional distinctiveness of grapes and wines, particularly in fermentations without the addition of exogenous yeasts. Several studies have demonstrated that certain fungi can persist in vineyards and be transmitted to grapes through grapevines [33,34,35,36]. Therefore, the origin and persistence of fungi have a crucial impact on wine quality and represent key indicators of stable regional characteristics in wine production.

## 5. Conclusions

To elucidate the key factors influencing wine regional characteristics, this study examined soil, must, and fermentation samples from Cabernet Sauvignon vineyards across five major Chinese wine regions. The results revealed significant regional differences in wine metabolite profiles, driven by the distinct microbial diversity of soils and wineries across different vineyards. Although the regional pattern of microbial flora changed during fermentation, the regional characteristics were retained at the end of fermentation. The contribution of microorganisms to wine aroma is closely related to their growth environments. Random forest and partial least squares path modeling analyses indicated that fungal diversity and weather conditions were key determinants of wine regional characteristics, with grape must fungal diversity being the most critical factor. In contrast, soil properties and soil bacterial diversity had relatively weak effects. Further analysis showed that weather and soil properties primarily influence wine metabolite profiles indirectly through their effects on soil and grape must microbial diversity, with this indirect influence outweighing the direct effects. In summary, this study provides new insights into the mechanisms underlying wine regional characteristics and offers practical guidance for enhancing wine quality through optimized vineyard management and targeted modulation of microbiota.

## Data Availability

The original contributions presented in this study are included in the article/Supplementary Material. Further inquiries can be directed to the corresponding author.

## Supplementary Materials

The following supporting information can be downloaded at: https://www.mdpi.com/article/10.3390/foods14071211/s1, Figure S1: Map of 5 sampling vineyards from four wine-producing regions in China, spanning 1500 km (east to west [E-W]). Figure S2: The rarefaction curve of the ITS2 region of fungi (a) and v3-v4 region of bacteria (b) in each sample under 97% similarity. Figure S3: Composition of soil fungal (a) and bacterial (b) communities in different vineyards. Figure S4: Changes in microbial diversity during wine fermentation. Table S1: Vineyard environmental conditions. Table S2: ANOVA results of the wine volatile compounds from each vineyard in 2022. Table S3: α-diversity (Shannon index) of soil and must microbiota from vineyards in five different regions.
