# Supplementary material for: The Fungal Microbiome in the Vineyard Ecosystem Plays a Key Role in Shaping the Regional Characteristics of Wine
Source: Foods. 2025 Mar 30;14(7):1211. doi: 10.3390/foods14071211 (PMC11989012; doi:10.3390/foods14071211)
Supplement: Supplementary file 1 [file foods-14-01211-s001.zip › foods-3510489-supplementary.pdf]

Supplementary material

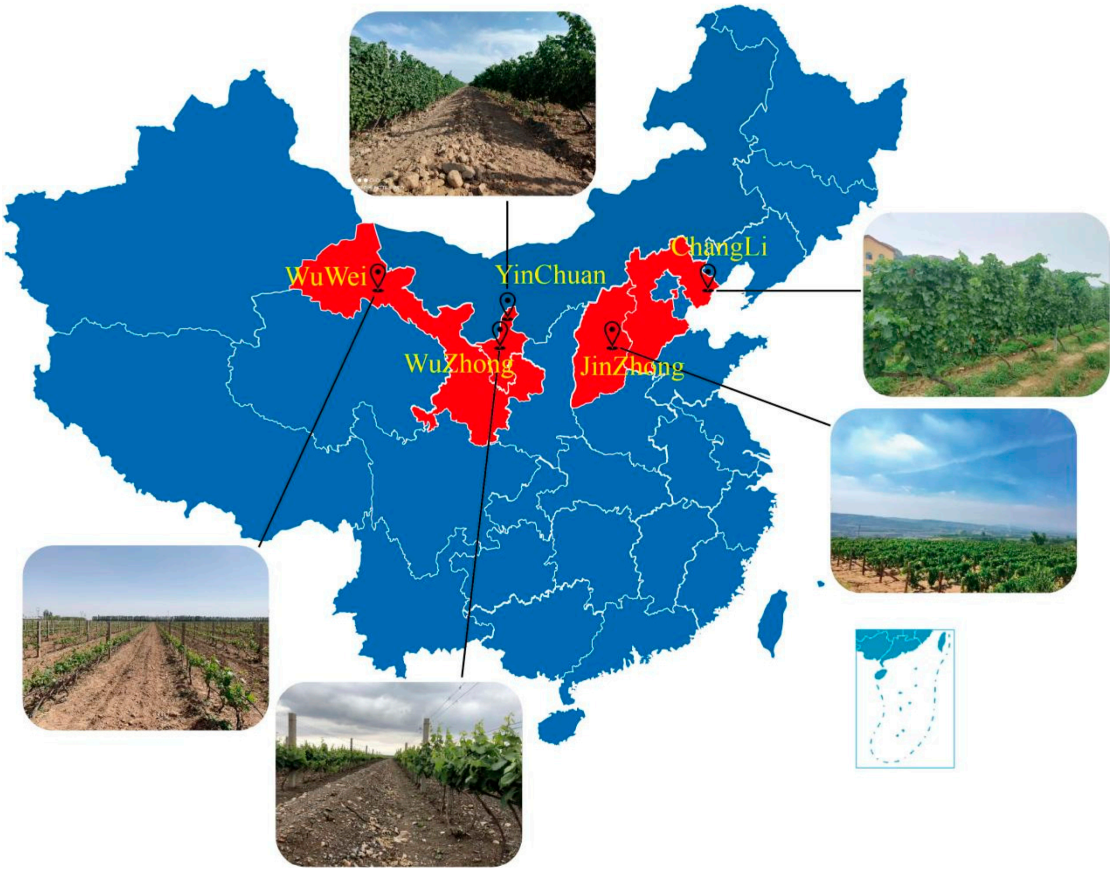

Figure S1 Map of 5 sampling vineyards from four wine-producing regions in China, spanning 1500 km (east to west [E-W]).

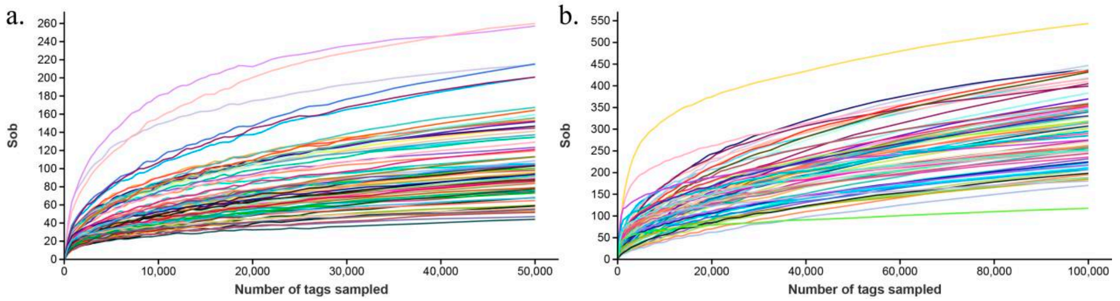

Figure S2 The rarefaction curve of the ITS2 region of fungi (a) and v3-v4 region of bacteria (b) in each sample under 97% similarity

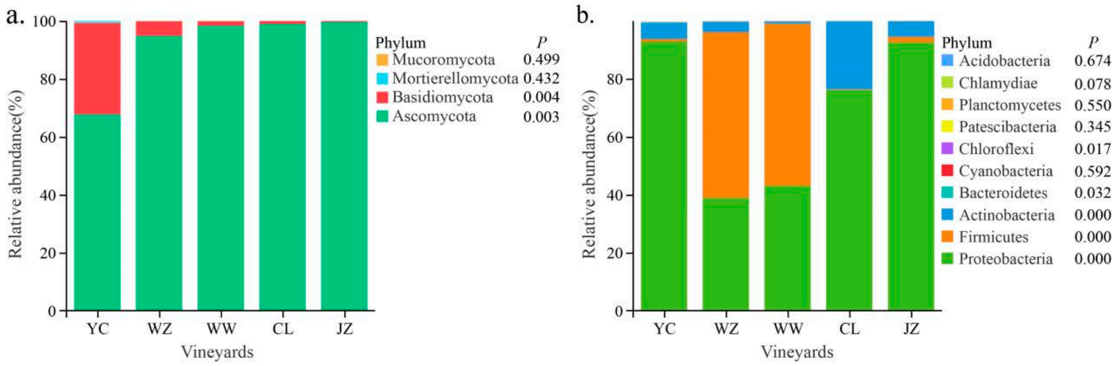

Figure S3 Composition of soil fungal (a) and bacterial (b) communities from different vineyards

Note: *P* values indicate whether each taxon is statistically significant between different vineyards.

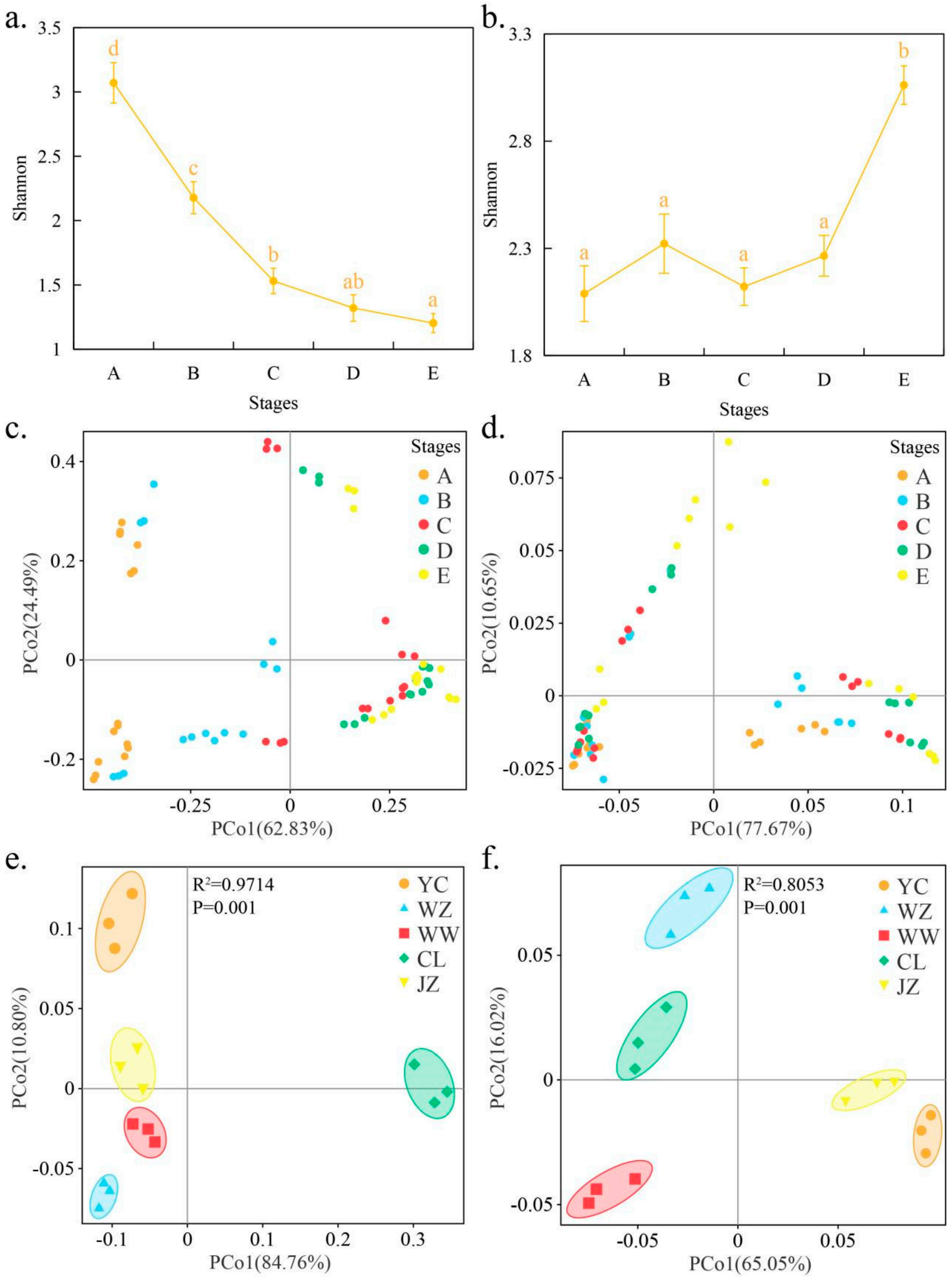

Figure S4 Changes in microbial diversity during wine fermentation.

Note: Changes in α-diversity (Shannon index) of fungi (a) and bacteria (b) during wine fermentation. PCoA analysis based on Bray-Curtis and weighted UniFrac distances showed the distribution patterns of fungal (c) and bacterial (d) communities

during fermentation. PCoA analysis based on Bray-Curtis and weighted UniFrac distances showed the distribution patterns of fungal (e) and bacterial (f) communities in the finished wine. A, at the must stage; B, at the onset of fermentation; C, during the early fermentation phase; D, during the mid-fermentation phase; E, at the end of fermentation. Different lowercase letters indicated the difference, with statistical significance ($p < 0.05$). A higher R^2 value indicates greater explained variance by grouping factors; a significance level of $P < 0.05$ was considered statistically significant.

Table S1 Vineyard environmental condition

Region	Altitude (m)	Annual average temperature (℃)	Annual precipitation (mm)	Sunshine duration (h)	Orientation	Soil texture	Cover crop	°Brix	Total acidity (g/L)
YinChuan	1100	10.6	182.6	2633	S	Sandy gravel	No cover	24.5	5.12
WuZhong	1100	11.2	209.3	2763	S	Sandy gravel	No cover	24.2	5.64
WuWei	1000	8.1	244.8	2737	S	Sandy loam	No cover	23.8	6.24
ChangLi	50	11.9	669.3	2679	S	Sandy loam	Grass	22.8	6.68
JinZhong	800	11.3	531.2	2548	S	Sandy loam	Grass	22.4	6.83

Table S2 ANOVA results of wine volatile compounds from each vineyard in 2022

Unit: μg/L

	Volatile Compound	CAS No.	YC	WZ	WW	CL	JZ	Pr (>F)
C1	Ethyl acetate	141-78-6	86399.91±1866.11	69247.69±2744.51	73867.68±1724.58	185205.36±2707.21	57282.93±944.32	***
C2	Ethyl isobutyrate	97-62-1	1.89±0.13	1.89±0.03	2.25±0.18	2.24±0.12	2.16±0.12	**
C3	Isobutyl acetate	110-19-0	63.36±2.71	58.66±2.07	111.13±3.89	124.35±9.73	107.26±1.17	*
C4	1-Propanol	71-23-8	1510.28±74.03	1521.01±114.14	923.27±62.21	1278.3±39.91	1132.26±98.46	***
C5	Ethyl butyrate	105-54-4	207.52±2.76	168.6±3.88	115.28±3.43	83.17±3.76	139.17±3.28	***
C6	Ethyl 2-Methylbutyrate	7452-79-1	4.11±0.11	3.83±0.11	1.52±0.02	0.98±0.12	1.74±0.14	***
C7	Ethyl isovalerate	108-64-5	2.77±0.23	2.72±0.59	0.35±0.03	0.17±0.01	0.2±0.06	*
C8	Isobutanol	78-83-1	55428.3±960.67	61348.67±859.78	66188±286.02	71985.92±532.68	62289.52±958.62	***
C9	Isopentyl acetate	123-92-2	2263.74±71.44	1740.28±87.15	1640.03±52.16	1203.37±93.59	1788.45±70.8	***
C10	1-Butanol	71-36-3	1296.12±33.03	2050.17±59.1	267.17±14.9	0±0	44.02±27.67	***
C11	D-Limonene	138-86-3	7.75±0.26	6.43±0.37	5.42±0.09	5.28±0.08	5.02±0.14	*
C12	3-Methyl-1-butanol	123-51-3	346592.98±9965.45	248026.51±682.88	234885.17±1471.67	217276.69±1423.99	212621.31±10432.48	***
C13	Ethyl hexanoate	123-66-0	236.28±4.73	97.75±19.55	105.77±12.54	9.28±8.78	235.17±8.06	***
C14	1-Pentanol	71-41-0	2.30±0.25	2.25±0.36	2.41±0.58	2.33±0.16	2.28±0.58	
C15	Hexyl acetate	142-92-7	6.98±0.14	7.15±0.43	10.96±0.57	14.33±0.97	13.46±0.41	***
C16	Octanal	124-13-0	0±0	8.26±0.29	4.42±0.76	0±0	2.73±0.86	**
C17	4-Methyl-1-pentanol	626-89-1	42.08±1.68	45.2±5.03	0±0	0±0	1.44±0.14	*
C18	3-Methyl-1-pentanol	589-35-5	102.34±2.47	109.63±3.58	0±0	0±0	0±0	**
C19	Ethyl lactate	97-64-3	520.29±59.57	790.06±23.76	7470.72±225.94	167.18±3.94	7971.85±47	***
C20	1-Hexanol	111-27-3	671.4±12.41	1119.21±84.06	850.98±4.71	2307.3±8.62	1060.36±57.91	***
C21	(-)-Rose oxide	3033-23-6	0.86±0.26	0.88±0.31	0.82±0.16	0.85±0.29	0.84±0.33	
C22	trans-3-Hexen-1-ol	928-97-2	14.39±0.92	29.5±2.83	18.62±0.29	32.9±1.9	27.83±0.59	***

C23	Leaf alcohol	928-96-1	15.99±0.84	25.37±2.96	39.74±3.58	30.96±0.67	28.62±0.75	***
C24	Methyl octanoate	111-11-5	1.08±0.05	0.96±0.10	0.91±0.03	0.67±0.10	0.98±0.07	**
C25	Nonanal	124-19-6	7.31±0.21	7.21±0.10	7.19±0.35	7.33±0.16	7.14±0.36	
C26	cis-2-Hexen-1-ol	928-94-9	0.59±0.22	0.61±0.32	0.56±0.35	0.62±0.35	0.60±0.36	
C27	Ethyl octanoate	106-32-1	0±0	0±0	210.21±15.36	65.02±5.52	276.23±7.88	***
C28	1-Octen-3-ol	3391-86-4	1.55±0.1	2.45±0.1	1.86±0.08	2.9±0.32	1.54±0.13	
C29	1-Heptanol	111-70-6	15.2±0.45	11.75±0.27	1.99±0.42	34.8±2.22	19.1±1.34	***
C30	Isoamyl caproate	2198-61-0	2.84±0.17	1.75±0.15	2.36±0.02	5.28±0.1	2.49±0.06	**
C31	Furfural	98--01-1	0.68±0.23	0.71±0.34	0.75±0.26	0.69±0.31	0.70±0.42	
C32	2-Ethylhexanol	104-76-7	098±0.03	0.94±0.10	0.93±0.06	0.97±0.10	0.96±0.08	
C33	Decanal	112-31-2	0.43±0.39	11.44±0.83	0±0	0.21±0.05	0.21±0.07	*
C34	Geraniol	106-24-1	10.7±0.47	12.11±1.17	10.4±0.11	14.15±4.48	16.7±0.56	*
C35	Ethyl 3-hydroxybutyrate	5405-41-4	239.73±13.39	249.64±22.22	114.2±14.34	0±0	152.23±7.36	***
C36	2-Isobutyl-3-methoxypyrazine	24683-00-9	0.52±0.22	0.51±0.32	0.53±0.35	0.52±0.35	0.52±0.36	
C37	Benzaldehyde	100-52-7	0.32±0.3	2.84±0.32	0.43±0.13	2.9±0.15	0±0	***
C38	Ethyl nonanoate	123-29-5	3.46±0.17	3.06±0.04	3.32±0.04	3.15±0.1	3.43±0.2	
C39	Propionic acid	79-09-4	779±36.16	1230.58±97.26	529.78±5.36	545.92±7.69	614.29±27.74	***
C40	Linalool	78-70-6	0.69±0.22	0.61±0.32	0.66±0.35	0.62±0.35	0.63±0.36	
C41	1-Octanol	111-87-5	3.01±0.28	2.52±0.05	3.32±0.01	19.5±0.44	13.85±0.5	***
C42	Isobutyric acid	79-31-2	2165.55±44.77	2734.51±76.15	2555.4±96.55	2449.15±47.82	2434.77±64.2	***
C43	Terpinen-4-ol	562-74-3	0±0	0.47±0.04	0±0	0±0	0.14±0.01	**
C44	Ethyl caprate	110-38-3	90.33±0.87	89.9±0.15	89.82±0	90.23±0.69	89.94±0.29	
C45	Phenylacetaldehyde	122-78-1	8.95±0.32	16.76±3.26	2.79±1.42	5.38±1.06	2.55±0.26	***
C46	Isovaleric acid	503-74-2	855.01±13.72	1174.96±54.53	365.78±9.24	331.21±10.91	383.06±10.74	*
C47	Diethyl succinate	123-25-1	3.11±0.17	3.16±0.14	3.12±0.14	3.15±0.15	3.13±0.21	
C48	α-Terpineol	98-55-5	0.81±0.04	0.78±0.04	0.74±0.01	1.24±0.14	1.21±0.48	*

C49	3-Methylthiopropanol	505-10-2	1677.18±49.93	1416.1±279.14	1017.84±51.4	834.6±33.27	1507.13±197.93	*
C50	Geranyl acetate	105-87-3	1.14±0.48	1.11±0.32	1.16±0.33	1.12±0.14	1.13±0.15	
C51	1-Decanol	112-30-1	097±0.08	0.91±0.05	0.96±0.02	1.02±0.14	0.95±0.05	
C52	Citronellol	106-22-9	0.56±0.08	0.58±0.04	0.53±0.01	0.56±0.06	0.56±0.04	
C53	Methyl salicylate	119-36-8	2.24±0.14	2.89±0.15	2.2±0.01	2.4±0.05	2.43±0.06	***
C54	Nerol	106-25-2	8.66±0.33	8.6±0.33	9.22±0.07	8.54±0.09	9.87±0.11	*
C55	Ethyl salicylate	118-61-6	2.02±0.09	2.07±0.07	2.18±0.01	2.21±0.02	2.48±0.08	***
C56	Phenethyl acetate	103-45-7	140.82±8.39	129.16±4.08	93.5±2.17	576.54±52.81	90.71±1.46	*
C57	Ethyl laurate	106-33-2	107.98±3.44	70.83±0.41	70.51±0.04	78.6±8.52	109.62±1.97	*
C58	Hexanoic acid	142-62-1	660.44±17.98	647.07±8.95	447.95±35.61	323.08±17.37	733.7±38.77	***
C59	Benzyl alcohol	100-51-6	274.91±12.42	426.83±67.94	302.19±20.17	351.78±10.14	166.78±15.3	*
C60	Whiskey lactone	39212-23-2	1.26±0.27	1.16±0.14	1.32±0.14	1.15±0.11	1.43±0.12	
C61	Phenethyl alcohol	60-12-8	79023.54±2344.81	101467.76±22856.16	49791.12±3157.94	44560.44±1052.65	46082.28±3433.84	*
C62	β-Lonone	79-77-6	1.07±0.08	1.01±0	1.06±0	1.11±0.04	1.1±0.05	
C63	Phenol	108-95-2	0±0	0.06±0.05	12.03±1.18	37.11±0.39	2.69±0.27	***
C64	4-Ethyl-2-methoxyphenol	2785-89-9	10.56±0.41	10.78±0.23	0±0	10.67±0.55	10.27±0.28	
C65	Octanoic acid	124-07-2	829.14±30.07	760.78±60.47	699.48±30.94	503.83±11	1033.14±65.82	***
C66	4-Ethylphenol	123-07-9	11.61±0.66	12.15±0.24	10.23±0.03	11.78±0.21	10.86±0.11	*
C67	Ethyl palmitate	628-97-7	136.76±11.27	248.89±32.41	106.44±4.04	56.57±2.76	63.41±2.11	**
C68	n-Decanoic acid	334-48-5	142.97±9.38	179.46±13.42	138.78±10.06	132.08±10.89	192.86±10.05	***
C69	Farnesol	4602-84-0	81.51±1.7	81.81±0.51	80.72±0.22	80.72±0.28	82.87±2.46	
C70	Benzoic acid	65-85-0	26.39±11.71	49.69±7.63	28.54±16.63	0±0	33.47±2.62	**

Note: Data are mean values of three independent samples ± standard deviation. Significance levels: *, $P < 0.05$; **, $P < 0.01$; ***, $P < 0.001$.

Table S3 α-diversity (Shannon index) of soil and must microbial communities from vineyards in five different regions

Region	Soil		Must	
	Bacteria	Fungi	Bacteria	Fungi
YC	3.275 ± 0.159	2.449 ± 0.101	2.147 ± 0.105	2.771 ± 0.105
WZ	3.422 ± 0.152	3.219 ± 0.285	1.976 ± 0.132	2.834 ± 0.107
WW	3.575 ± 0.129	2.701 ± 0.392	1.667 ± 0.113	2.614 ± 0.151
CL	3.104 ± 0.146	2.307 ± 0.261	2.460 ± 0.126	3.123 ± 0.117
JZ	3.911 ± 0.110	2.043 ± 0.105	2.196 ± 0.157	4.004 ± 0.171

Data are mean values of three independent samples ± standard deviation.
